# Role of Bioaerosols on the Short-Distance Transmission of Multidrug-Resistant Methicillin-Resistant *Staphylococcus aureus* (MRSA) in a Chicken Farm Environment

**DOI:** 10.3390/antibiotics11010081

**Published:** 2022-01-10

**Authors:** Bing-Mu Hsu, Jung-Sheng Chen, Gwo-Jong Hsu, Suprokash Koner, Viji Nagarajan, Hsin-Chi Tsai

**Affiliations:** 1 Department of Earth and Environmental Sciences, National Chung Cheng University, Chiayi 621, Taiwan; bmhsu@eq.ccu.edu.tw (B.-M.H.); suprokashkoner22@gmail.com (S.K.); mathumitha08@gmail.com (V.N.); 2 Department of Medical Research, E-Da Hospital, Kaohsiung 824, Taiwan; nicky071214@gmail.com; 3 Division of Infectious Diseases, Ditmanson Medical Foundation, Chia-Yi Christian Hospital, Chiayi 600, Taiwan; cych01347@gmail.com; 4 Department of Biomedical Sciences, National Chung Cheng University, Chiayi 621, Taiwan; 5 Department of Psychiatry, School of Medicine, Tzu Chi University, Hualien 970, Taiwan; 6 Department of Psychiatry, Tzu Chi General Hospital, Hualien 970, Taiwan

**Keywords:** methicillin-resistant *Staphylococcus aureus* (MRSA), bioaerosol transmission, genotyping, antimicrobial resistance, toxins

## Abstract

Methicillin-resistant *Staphylococcus aureus* (MRSA) is a dynamic and tenacious pathogenic bacterium which is prevalent in livestock farming environments. This study investigated the possibility of MRSA spread via bioaerosol transmission from an indoor chicken farm environment to outdoors downwind (up to 50 m). The concentration of total airborne bacteria colony formation units (CFUs) was decreased with increasing sampling distance ranging from 9.18 × 10^1^ to 3.67 × 10^3^ per air volume (m^3^). Among the 21 MRSA isolates, 15 were isolated from indoor chicken sheds and exposure square areas, whereas 6 were isolated from downwind bioaerosol samples. Molecular characterization revealed that all of them carried the staphylococcal cassette chromosome *mec* (SCC*mec*) VIII, and they were remarkably linked with the hospital-associated MRSA group. *Spa* typing analysis determined that all MRSA isolates belonged to *spa* type t002. Virulence analysis showed that 100% of total isolates possessed exfoliative toxin A (*eta*), whereas 38.09% and 23.80% strains carried exfoliative toxin B (*etb*) and enterotoxin A (*entA*). Additionally, all of these MRSA isolates carried multidrug resistance properties and showed their resistance against chloramphenicol, ciprofloxacin, clindamycin, tetracycline, and erythromycin. In addition, chi-squared statistical analysis displayed a significant distributional relationship of gene phenotypes between MRSA isolates from chicken farm indoor and downwind bioaerosol samples. The results of this study revealed that chicken farm indoor air might act as a hotspot of MRSA local community-level outbreak, wherein the short-distance dispersal of MRSA could be supported by bioaerosols.

## 1. Introduction

Antibiotic-resistant strains of Gram-positive pathogens, especially *S. aureus*, have been isolated from diverse environments such as hospitals, long-term care facilities, rivers, sediments, lakes, soil, and even the deep ocean [1,2]. However, bioaerosol-associated risk related to *S. aureus* remains to be fully understood. The dispersion of microorganisms comprising the pathogenic bacteria associated with bioaerosols can spread in different environments due to their highly aerodynamic properties, such as small diameter and lightweight [3]. Several occupational units (food processing, livestock, waste dumping station, agricultural farmland) and human activities (coughing, sneezing, washroom, and floor cleaning) are important sources of bioaerosol formation in the air [4,5]. Kabelitz et al. [6] had mentioned that due to extensive livestock production, the concentration of bioaerosols could increase downwind of farming areas. A better understanding of bioaerosols’ functions and the characterization of existing pathogenic microbes could help in estimating the risk of infection in humans and livestock populations by such pathogens [7]. Kim et al. [8] reported that in most zoogenic bacteria and virus-related infectious diseases, such as anthrax, Q-fever, avian and swine influenza, and brucellosis, their severity is propagated via bioaerosol exposure in the environment.

*Staphylococcus aureus* (*S. aureus*) is a highly prevalent pathogenic airborne microbe in livestock farming environments [9]. Its severity could increase by acquiring multidrug resistance properties which causes a serious health threat in a host. For example, methicillin-resistant *S. aureus* (MRSA) has enough potential to exhibit nosocomial infections in human and livestock bodies [10]. The surveillance on how MRSA clones are transmitting from one environment to another and their proliferation in a particular area is critical to set up the control strategies against them. Previous studies have shown that the bioaerosols of indoor air in both livestock farming and hospital areas could carry MRSA strains and possibly act as a hotspot for spreading MRSA in the environment [10,11]. MRSA infection was primarily limited to persons directly associated with healthcare facilities with poor immune systems, which is called hospital-acquired MRSA (HA-MRSA) infection [12]. Later on, community-associated MRSA (CA-MRSA) infection was prevalent in healthy people who had never been hospitalized before. Additionally, MRSA strains which are associated with the infection of animal farming and food processing units are described as livestock-associated MRSA (LA-MRSA) [13].

Strain characterization and genotyping are typically required for MRSA epidemiological study. Here, *mecA* containing the staphylococcal cassette chromosome *mec* (SCC*mec*) element is considered a biomarker to identify whether the *S. aureus* strain belongs to MRSA or not [14]. Translation of this *mecA* gene can produce PBP2a protein (penicillin-binding protein 2a), which is mainly responsible for gaining methicillin resistance properties in the *S. aureus* strain [2]. Furthermore, based on the *mec* and *ccr* complex sequences in the SCC*mec* element, it can be classified into I–XI types [2,15]. On the other hand, among different kinds of methods to monitor the epidemiology of MRSA, such as pulsed field gel electrophoresis (PFGE), biotyping, *coa* typing, prophage typing, multi-locus sequence typing (MLST), *spa* typing is the most cost-effective, least time consuming, and robust method [15]. The X region of the *spa* gene of the MRSA whole genome has been focused to perform *spa* typing analysis [16]. In addition, the production of various toxin factors such as enterotoxin, exotoxin, and exfoliative toxins (*ETs*) by MRSA strains has been linked to staphylococcal food poisoning, scalded skin syndrome, and toxic shock syndrome toxin (*TSST*) [17].

Recently, the MRSA strain has been designated as the most concerning emerging pathogenic threat in different types of livestock farming units, posing a serious health and safety issue for both farmworkers and livestock regarding safe production [18,19,20]. In this context, our past study identified that bioaerosols of poultry farm indoor air are a suitable carrier of MRSA colonies [11]. However, a detailed view pattern of MRSA strain transmission and distance spread via bioaerosol from poultry farming sources and epidemiology remains unclear [9,21,22]. The present study investigated the role of bioaerosol dispersal in transmitting MRSA strains from commercial chicken farm areas to the surrounding environment by using a molecular typing approach. The SCC*mec* element was amplified for categorization of the isolated MRSA strains. *Spa* typing was conducted to describe their epidemiology, whereas virulence factors were targeted to identify their pathogenicity. Additionally, an antimicrobial drug resistance test was conducted to evaluate their multidrug resistance pattern.

## 2. Results

### 2.1. Odor Compounds and Airborne Bacteria Load in Chicken Farm Ambient Air

To obtain the background information on the dispersal of odor compounds in the ambient air from chicken farming practices, the concentrations of ammonia, methylamine, hydrogen sulfide, and mercaptan were estimated for every sampling point. The presence of odor-producing compounds such as ammonia and methylamine were only found in the first and second chicken shed, including the exposure square area of ambient air (Table 1). Ammonia and methylamine concentrations varied between 1.5 and 4.5 ppm and 0.5 and 2.5 ppm per air volume (meter cube), respectively. However, the concentration of such odor-producing compounds was below the detection limit in downwind (5–50 m) and upwind (up to −50 m) sampling points. The dominant wind was blowing from the north-east and north-west directions of the chicken farm, and the wind speed ranged from 0 to 3 m/s. The concentration of total airborne bacteria varied from 9.18 × 10^1^ to 3.67× 10^3^ CFU per volume air (meter cube) all over the air samples, as shown in Table 1. The indoor air samples from the chicken farm and exposure square had much higher bacteria content (5.75 × 10^2^–3.67 × 10^3^ CFU) than the downwind sampling point (9.18 × 10^1^–1.40 × 10^3^ CFU). The concentration of airborne bacteria continuously decreased with the increasing distance of sampling points (up to 50 m) from the source point (chicken farm). The lowest concentration of airborne bacteria was observed in the upward wind sampling point. In addition, the Pearson correlation coefficient value showed that the concentration of total bacterial CFU had a highly positive correlation (*p* = 0.01) with the concentration of ammonia and methylamine in bioaerosol samples, as shown in Table S3. All of these sampling points carried MRSA in the air, except the 20 m downwind and 50 m upwind sampling points. However, bioaerosol samples associated with chicken farm indoor and exposure areas had higher amounts of MRSA. The concentration of MRSA in bioaerosols was decreased with increasing the sampling distance, associated with downwind samples in this order 3 > 5 > 50 m.

### 2.2. Occurrence of SCCmec Bearing MRSA Clone Bearing and Their Spa Typing in Bioaerosol Samples 

A total of 21 pure MRSA isolates were isolated from bioaerosol samples associated with the majority of sampling points, except for the 20 m downwind and 50 m upwind sampling point. All the MRSA isolates and their Id numbers are shown in Table 2. The bioaerosol samples from the chicken shed indoor air and exposure area carried 15 MRSA isolates, where 3, 5 and 50 m downwind samples carried 3, 2, and 1 isolates, respectively. However, strain categorization revealed that all of these isolates carried the SCC*mec* element type VIII and belonged to the hospital-associated MRSA group. Additionally, none of them carried the PVL gene. The *Spa* typing analysis further revealed that these isolates belonged to one spa type (t0002). The chi-squared test score demonstrated that all the isolated MRSA isolates from different sampling points were associated with each other in terms of SCC*mec* typing, *spa* typing and belonged to the hospital-associated MRSA group, as shown in Table S5.

### 2.3. The Profiling of Virulence Factor Encoding Genes for Isolated MRSA Clones 

The virulence-factor-associated genes profiling result showed that 23.80% and 100% of total MRSA isolates from chicken farm environment bioaerosol samples carried enterotoxin gene *entA* and exfoliative toxin gene *eta*, as shown in Table 3 and Table 4. In contrast, 38.09% of isolates carried the exfoliative toxin gene *etb*. However, none of them carried exotoxin genes such as *tsst-1*. The exfoliative toxin gene *etb* was mostly found in the isolates from chicken shed 2 and the exposure square. In the case of enterotoxin gene *entA*, this was mainly identified from the exposure square and downwind (3, 5 and 50 m) distance sampling point MRSA isolates. The exfoliative toxin gene *eta* was found in all the isolated MRSA clones from each sampling point of this study.

### 2.4. Antimicrobial Property and Multidrug Resistance Pattern of Isolated MRSA Clones

The results of the antimicrobial susceptibility test against eight selected antibiotics (clindamycin, gentamicin, sulfamethoxazole–trimethoprim, chloramphenicol, tetracycline, erythromycin, ciprofloxacin, rifampicin) on 21 MRSA isolates of this study are shown in Table 4 and Table 5. According to the diameter of the zone of inhibition, all MRSA isolates from chicken shed indoor air, the exposure area and downwind (3, 5 and 50 m) distance bioaerosol samples were resistant to chloramphenicol, ciprofloxacin, clindamycin, and tetracycline. At the same time, 95.23% of total isolates were resistant to erythromycin. However, none of them were resistant to gentamicin, rifampicin, and sulfamethoxazole–trimethoprim. Detailed standard scores for the zone of inhibition diameter measurements to determine the resistance in MRSA isolates are shown in supplementary Table S4. A total of twenty isolates showed the MDR pattern belonging to five groups of antibiotics (C-CIP-CC-E-T), and only one isolate displayed the MDR pattern belonging to four groups of antibiotics (C-CIP-CC-T). Overall, all MRSA isolates exhibited multiple drug resistance properties.

## 3. Discussion

Previous studies related to pathogenic microbes highlighted that diverse Gram-positive and Gram-negative bacteria such as *S. aureus*, *Escherichia coli*, *Streptococcus suis*, and *Actinobacillus pleuropneumoniae* are most prevalent in livestock farming bioaerosol samples [23,24]. The loads of viable bacterial colony formation units in this study were much higher in the first and second chicken sheds, and the exposure square area samples than downwind bioaerosol samples, as shown in Table 1. Gibbs et al. [25] reported a similar trend in decreased airborne bacteria CFUs in downwind sampling zones up to 150 m distance from the swine barn area, which is consistent with our results. Additionally, Liu et al. demonstrated the transmission of *S. aureus* in the air from henhouse indoor to outdoor downwind sampling areas [9]. In this study, the MRSA strain was also isolated from both chicken sheds and downwind sampling points (up to 50 m), which is in line with our previous study results [11]. Therefore, it could be suggested that the bioaerosol samples in the present study had adequate potential to carry and transmit the MRSA strains from the chicken shed indoor air to the surrounding downwind ambient environment. In addition, the number of total MRSA isolates colonies was continuously decreased with increasing the sampling distance from the chicken farm indoor environment. According to a previous study, the load of airborne bacteria in indoor and outdoor bioaerosol might vary due to the air exchangeable rate and carbon dioxide concentration of ambient air [26]. Additionally, Homidan et al. [27] have discussed that ammonia concentrations in poultry farm indoor air might be influenced by the poor ventilation system, it may directly reduce the air exchangeable rate of chicken farm indoor environment which help to increase the CFU load in the air. In our study, a similar trend was observed using statistical analysis. For example, the correlation coefficient (r) value showed that the prevalence of MRSA and viable airborne bacteria CFU load was negatively correlated with wind speed and positively correlated with ammonia and methylamine concentrations (Supplementary Table S3). The outdoor bioaerosol sampling area had a high wind speed; therefore, there was an enhanced air exchangeable rate. This factor might elevate more bioaerosol dispersal and negatively impact the CFU count of total bacteria, specifically, the MRSA prevalence in outdoor downwind bioaerosol samples (up to 50 m) of this study. The Pearson correlation coefficient R-value also showed a negative relationship between the total bacteria count from bioaerosol samples and the distance of sampling point in the downwind area (Supplementary Table S3). This result could elaborate that in an outdoor environment, increasing the sampling distance might positively influence the air exchangeable rate. As the air exchangeable rate increases, it can downregulate the CFU load. Additionally, the occurrence of ammonia and methylamine may be associated with the CFU count of airborne bacteria and MRSA load in chicken sheds and exposure area bioaerosol samples of this study [28].

Butaye et al. [24] have described that the SCC*mec* (type III, IV and V) containing LA-MRSA (CC9 strain) are mainly found from different livestock units of many Asian countries such as Thailand, China, Hong Kong, Malaysia, Taiwan, and South Korea. All the isolated MRSA isolates from eight bioaerosol samples of this study carried the SCC*mec* type VIII elements and belonged to hospital-associated MRSA group. This finding is in line with the previous study results of bioaerosol samples associated with chicken farm environments [11]. Funaki et al. [29] demonstrated that SCC*mec* type VIII could also be found in community harbored MRSA strains. An Asia-specific report highlighted that most HA-MRSA isolates in Taiwan carried SCC*mec* elements type IV and III [30]. The identified SCC*mec* type VIII element bearing MRSA isolates in this study possibly belonged to a new lineage of epidemic MRSA strain, according to the International Working Group on the Classification of Staphylococcal Cassette Chromosome Elements (IWG-SCC). The existence of SCC*mec* type VIII element in MRSA strains was initially reported in Canada, and it was also later found in the United States [31,32]. Additionally, a previous report revealed that *spa* type t002 was one of the most prevalent MRSA strains in Asia, Europe, and America [33]. In this present study, all the MRSA isolates belonged to the t002 *spa* type, which is consistent with the results of past studies from bioaerosols and environmental samples associated with chicken and swine farming areas [11,34]. Therefore, we suggest that MRSA isolates in the air samples associated with indoor and outdoor downwind (up to 50 m) chicken farm areas in this study have enough potential to causes outbreaks of epidemic nosocomial infection in the surrounding environment via bioaerosol transmission.

MRSA constitutes several virulence factors that determine the severity of their pathogenesis in the target host. Among several virulence factors, such as exotoxin, enterotoxin, and exfoliative toxin-encoding genes only the *eta* was positive for all the isolates of this study. In contrast, 38.09% and 23.80% of total isolates were positive for the *etb* and *entA* genes. A past study showed 100% and 70.2% detection rates of *eta* and *etb* genes all over the MRSA isolates from poultry farm bioaerosol samples, respectively, which is consistent with the results of the present study [11]. However, Szafraniec et al. [35] demonstrated that most exfoliative toxin *A* gene infections in chickens were related to *Staphylococcus hyicus.* Pathogenicity by exfoliative toxin genes *eta* and *etb* had much greater severity on the human body by stimulating skin peeling and the blistering development in the host cell [36,37,38]. However, these exfoliative toxin genes, *eta* and *etb*, could also develop staphylococcal pathogenicity on other livestock bodies, e.g., porcine [39]. Additionally, enterotoxin gene A (*entA*) detection in this study could highlight that the MRSA isolates might have the potential to produce a high level of super antigenic activity, such as through inflammatory cytokines via the disruption of adaptive immunity through stimulating T cells [40]. Thus, we suggest that infected hosts might face the severe pathogenicity of MRSA during staphylococcal outbreaks from chicken farm bioaerosols to adjacent air.

Due to the misuse and overuse of various antibiotics, resident microorganisms in livestock farming may acquire antimicrobial resistance properties via selective pressure [41]. Past studies on antibiotic resistance associated with airborne microbes from livestock farming areas had described that tetracycline- and erythromycin-resistant *S. aureus* was the most prevalent pathogenic bacteria in this environment, posing a serious risk to public health [41,42]. In this study, 100% and 95.23% of the total MRSA isolates from chicken farm indoor and outdoor samples were resistant to chloramphenicol, ciprofloxacin, clindamycin, tetracycline, and erythromycin. At the same time, all of them exhibited multiple drug resistance properties based on the disk diffusion method. In this context, Tao et al. [11] have observed a similar antibiotic resistance pattern in MRSA isolates associated with chicken farm bioaerosol samples, which is consistent with the present study. Similarly, Liu et al. [9] demonstrated that *S. aureus* transmission via bioaerosol from an indoor poultry farm environment to downwind air was resistant to sulfamethoxazole, penicillin, tetracycline, chloramphenicol and erythromycin, which also supports our study results. Additionally, the molecular characterization of MRSA isolates following the chi-squared statistical test revealed that all 21 isolates from each sampling area were genotypically associated with each other (Supplementary Table S5). Therefore, the current investigation demonstrates a multidrug-resistant MRSA strain population that could be an emerging epidemiological risk near chicken farms, which warrants thorough pathogen management and control measures.

## 4. Materials and Methods

### 4.1. Sampling Information and Site Description

**Figure 1:**
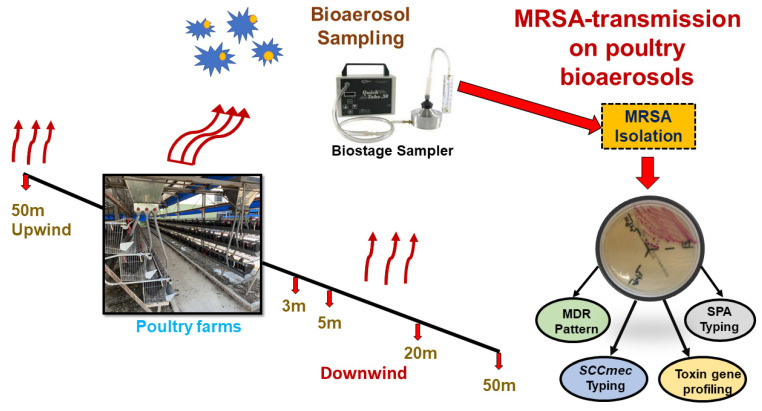
Schematic diagram of study design and chicken farm bioaerosol sampling strategy.

The sampling site of this present study was in Dalin county of southern Taiwan. The primary objective of this sampling was to survey the pattern of MRSA colonies spread out from the chicken farm indoor environment to the outdoor environment via bioaerosol transmission and including their molecular typing. A total of 8 bioaerosol samples were collected from two adjacent chicken sheds inside, one exposure square (an empty place between these two chicken sheds), one upwind and four downwind (up to 50 m) area’s ambient air. Geographical coordinates of each sampling point have been described in supplementary Table S1. The procedure of bioaerosol samples collection was done by following the previous study’s standardized protocol of bioaerosol collection [11]. Figure 1 shows the overview of the bioaerosol sampling strategy and molecular typing of MRSA isolates.

### 4.2. Bacterial Colony Capturing Method from Air and Environmental Parameters Analysis 

Specific CHROMagar™ MRSA was inserted into the BioStage sampler to capture the existed MRSA isolates from bioaerosol samples. In addition, tryptic soy agar (TSA) with 100 mg/mL cycloheximide was used according to a prior study protocol to count the overall bacteria colony formation unit per volume of air samples (meter cubed) [11,43]. The direction and speed of the wind were detected using a digital anemometer (Puxicoo P6-8232). Additionally, the concentrations of ammonia, mercaptans, methylamine, and hydrogen sulfide in the surrounding ambient air was measured to show the odor production by the chicken shed. A gas detector system and detection tubes (Gastec Inc., Fukayanaka, Japan) were used to measure the concentration of these gaseous compounds in the ambient air, according to the instructions of the company provided in the operating manual (https://www.gastec.co.jp/en/instructionmanual, accessed on 6 June 2020).

### 4.3. Isolation and Culture of MRSA Isolates from Bioaerosol Samples

After sampling the CHROMagar™ MRSA was pulled out from the BioStage sampler and directly inserted into an incubator at 37 °C for 24 h to let the MRSA colony grow properly. Next, to isolate the MRSA in pure culture, the mauve color grown colony was picked up and put into brain–heart infusion broth (BHIB) media following incubation at 37 °C temperature for 24 h. Again, one loop of BHIB was taken up and streaked on a Baird–Parker agar plate following incubation at 37 °C for 24 h. Finally, a pure single MRSA colony was inoculated into BHIB and grown for 24 h at 37 °C. The next day, the grown culture of MRSA was preserved in 33% glycerol at −20 °C. Later, this pure cultured broth was used for DNA extraction.

### 4.4. Molecular Typing of MRSA Isolates

Genomic DNA of MRSA was extracted using a commercial DNA extraction kit (MagPurix Bacterial DNA Extraction Kit, ZP02006), following the protocol described in previous studies [2,11]. After that, different molecular typing experiments were performed via PCR analysis to characterize the MRSA isolates. Briefly, *Nuc* and *mecA* gene sequence was amplified to confirm the isolated colonies from samples belonging to MRSA strain. SCC*mec* element and the panton–valentine leukocidine (*PVL*) gene were targeted to categorize MRSA isolates. Three toxin genes, including exfoliative toxins (*eta* and *etb*), enterotoxins (*ent A* to *E*), and exotoxin gene toxic shock syndrome toxin-1 (*tsst-1*), were targeted to determine their virulence factors. Additionally, in this study, *spa* typing was conducted using commercial software (BioNumerics) to describe the epidemiological aspect of MRSA isolates. The primers sequences used to identify these genes, along with the respective PCR reaction protocols, have been provided in supplementary Table S2. Electrophoresis analysis was performed on 1.5% agarose gel at 110 V for 30 min to confirm the presence of the respective PCR product in each PCR amplification.

### 4.5. Anti-Microbial Susceptibility Test

A total of 8 antibiotics—clindamycin (DA, 2 µg), gentamicin (G, 10 µg), sulfamethoxazole-trimethoprim (S/T, 23.75/1.75 µg), chloramphenicol (C, 30 µg), tetracycline (T, 30 µg), erythromycin (E, 15 µg), ciprofloxacin (CIP, 5 µg), and rifampicin (RA, 5 µg)—were selected to determine the antimicrobial resistance properties of MRSA isolates using the disk diffusion method, as described previously [11,44]. The multidrug resistance pattern of MRSA isolates was categorized as previously described by Magiorakos et al. [45]. A standard score of the zones of inhibition diameter of reported antibiotic-resistant strain has been shown in Supplementary Table S4, which was used to characterize the antibiotic resistance of MRSA isolates. The standard scores of the zones of inhibition diameters for the applied antibiotics were determined according to the documents from the Clinical & Laboratory Standards Institute (CLSI) [46].

### 4.6. Statistical Analysis

After checking the linear distribution of the data, Pearson correlation analysis was performed to determine the impact of environmental parameters on the load of airborne bacteria colony count using SPSS software (IBM SPSS statistics 24). The chi-squared test was also performed with SPSS software to prove the distributional relationship between MRSA isolates from indoor and outdoor chicken farm bioaerosol samples.

## 5. Conclusions

The indoor environment of the chicken sheds of this study could propagate the airborne bacteria, wherein MRSA colonies could spread out from chicken sheds indoor to outdoor environments via bioaerosol transmission. The wind speed and direction may determine their dispersal patterns in the ambient air of chicken farms. At this point, the MRSA colonies from the indoor chicken shed environments were mostly transmitted to the exposure square and downwind areas of chicken sheds. Our strain characterization results highlighted that all the MRSA isolates contained SCC*mec* element type VIII and fitted into spa type t002 and hospital-harbored MRSA strain. All (100%) of the isolates carried exfoliative toxin gene A (*eta*), 38.09% carried exfoliative toxin gene B (*etb*), and 23.80% carried enterotoxin gene class A (*entA*). These isolates possess drug resistance properties against chloramphenicol, ciprofloxacin, clindamycin, tetracycline, and erythromycin. The findings of this study highlighted strong evidence on MRSA strain transmission from chicken sheds indoor air to downwind outdoor air. In addition, the molecular typing results underpinned the elevated epidemiological risk by exposing humans and livestock to pathogenic hospital-associated MRSA. Multi-drug resistance characteristics of MRSA isolates could trigger a devastating nearby-community level human as well as the livestock health risk that could be difficult to control.

## Figures and Tables

**Table 1:** The concentration of odor-producing compounds and the total airborne bacteria in the ambient air.

Sampling Point	Concentration of Odor Pollutants (ppm)				Wind Direction	Wind Speed (m/s)	Total Bacteria Count	MRSA Prevalence
	Ammonia	Methylamine	Hydrogen Sulfide	Mercaptan			CFU/m^3^ (by Biostage)	
1st chicken shed	3	2.5	<LOD	<LOD	North-east	0.4–0.5	3.67 × 10^3^	5
2nd chicken shed	4.5	2.5	<LOD	<LOD	North-west	0.5–1.2	3.33 × 10^3^	5
Exposure square	1.5	0.5	<LOD	<LOD	South-east	0–0.4	5.75 × 10^2^	5
3 m Downwind	<LOD	<LOD	<LOD	<LOD	North-east	0.9–1.5	1.40 × 10^3^	3
5 m Downwind	<LOD	<LOD	<LOD	<LOD	North-east	0.6–1.0	9.75 × 10^2^	2
20 m Downwind	<LOD	<LOD	<LOD	<LOD	South-west	1.4–1.6	3.03 × 10^2^	ND
50 m Downwind	<LOD	<LOD	<LOD	<LOD	South-east	0.6–1.4	2.89 × 10^2^	1
50 m Upwind	<LOD	<LOD	<LOD	<LOD	North-west	2.4–3	9.18 × 10^1^	ND

LOD = lower than detection limit (ammonia ≤ 0.5 ppm, hydrogen sulfide ≤ 0.05 ppm, methylamine ≤ 0.01 ppm, mercaptan = 0.01 ppm); ND = not detected (sample has no MRSA isolates).

**Table 2:** SCC*mec* and *spa* typing outcome of MRSA isolates.

Sampling Point	MRSA Isolates Number	MRSA Isolates ID	SCC*mec* Typing			*Spa* Typing
			SCC*mec*	PVL	HA, CA, LA	
1st chicken shed	1	JCYB101	VIII	-	HA	t002
	2	JCYB102	VIII	-	HA	t002
	3	JCYB103	VIII	-	HA	t002
	4	JCYB104	VIII	-	HA	t002
	5	JCYB105	VIII	-	HA	t002
2nd chicken shed	6	JCYB201	VIII	-	HA	t002
	7	JCYB202	VIII	-	HA	t002
	8	JCYB203	VIII	-	HA	t002
	9	JCYB204	VIII	-	HA	t002
	10	JCYB205	VIII	-	HA	t002
Exposure square	11	JCYB301	VIII	-	HA	t002
	12	JCYB302	VIII	-	HA	t002
	13	JCYB303	VIII	-	HA	t002
	14	JCYB304	VIII	-	HA	t002
	15	JCYB305	VIII	-	HA	t002
3 m Downwind	16	JCYB401	VIII	-	HA	t002
	17	JCYB402	VIII	-	HA	t002
	18	JCYB403	VIII	-	HA	t002
5 m Downwind	19	JCYB501	VIII	-	HA	t002
	20	JCYB502	VIII	-	HA	t002
20 m Downwind	ND	ND	ND	ND	ND	ND
50 m Downwind	21	JCYB701	VIII	-	HA	t002
50 m Upwind	ND	ND	ND	ND	ND	ND

ND = not detected (sample has no MRSA isolates).

**Table 3:** Virulence factors profiling results of MRSA isolates.

Virulence Factors	1st Chicken Shed	2nd Chicken Shed	Exposure Square	3m Downwind	5m Downwind	20m Downwind	50m Downwind	50m Upwind	Overall Detection Rate (%)
	MRSA Isolates (*n* = 5)	MRSA Isolates (*n* = 5)	MRSA Isolates (*n* = 5)	MRSA Isolates (*n* = 3)	MRSA Isolates (*n* = 2)	MRSA Isolates (*n* = 0)	MRSA Isolates (*n* = 1)	MRSA Isolates (*n* = 0)	Total MRSA Isolates (*n* = 21)
*entA*	(0\5)	(0\5)	(1\5)	(2\3)	(2\2)	ND	(1\1)	ND	23.80%
*entB*	(0\5)	(0\5)	(0\5)	(0\3)	(0\2)	ND	(0\1)	ND	0%
*entC*	(0\5)	(0\5)	(0\5)	(0\3)	(0\2)	ND	(0\1)	ND	0%
*entD*	(0\5)	(0\5)	(0\5)	(0\3)	(0\2)	ND	(0\1)	ND	0%
*entE*	(0\5)	(0\5)	(0\5)	(0\3)	(0\2)	ND	(0\1)	ND	0%
*eta*	(5\5)	(5\5)	(5\5)	(3\3)	(2\2)	ND	(1\1)	ND	100%
*etb*	(0\5)	(4\5)	(4\5)	(0\3)	(0\2)	ND	(0\1)	ND	38.09%
*tsst-1*	(0\5)	(0\5)	(0\5)	(0\3)	(0\2)	ND	(0\1)	ND	0%

ND = not detected (sample has no MRSA isolates).

**Table 4:** The virulence gene identification and antibiotic resistance patterns of every single MRSA isolate.

Sampling Point	Numbers of MRSA Isolates	Detection of Virulence Genes	Antibiotic Resistance
First chicken shed	1	*eta*	C-CIP-CC-E-T
	2	*eta*	C-CIP-CC-E-T
	3	*eta*	C-CIP-CC-E-T
	4	*eta*	C-CIP-CC-E-T
	5	*eta*	C-CIP-CC-E-T
Second chicken shed	6	*eta*, *etb*	C-CIP-CC-E-T
	7	*eta*, *etb*	C-CIP-CC-E-T
	8	*eta*, *etb*	C-CIP-CC-E-T
	9	*eta*, *etb*	C-CIP-CC-T
	10	*eta*	C-CIP-CC-E-T
Exposure square	11	*eta*, *etb*	C-CIP-CC-E-T
	12	*eta*, *etb*	C-CIP-CC-E-T
	13	*eta*, *etb*	C-CIP-CC-E-T
	14	*eta*, *etb*	C-CIP-CC-E-T
	15	*entA*, *eta*	C-CIP-CC-E-T
3 m Downwind	16	*eta*	C-CIP-CC-E-T
	17	*entA*, *eta*	C-CIP-CC-E-T
	18	*entA*, *eta*	C-CIP-CC-E-T
5 m Downwind	19	*entA*, *eta*	C-CIP-CC-E-T
	20	*entA*, *eta*	C-CIP-CC-E-T
20 m Downwind	ND	ND	ND
50 m Downwind	21	*entA*, *eta*	C-CIP-CC-E-T
50 m Upwind	ND	ND	ND

*eta* = exfoliative toxin gene A; *etb =* exfoliative toxin gene B; *entA* = enterotoxin gene A; C-CIP-CC-E-T = chloramphenicol–ciprofloxacin–clindamycin–erythromycin–tetracycline; C-CIP-CC-T = chloramphenicol–ciprofloxacin–clindamycin–tetracycline; ND = not detected (sample has no MRSA isolates).

**Table 5:** The value of zone of inhibition diameter measurements and multiple drug resistance categorization of MRSA isolates.

	Chloramphenicol	Ciprofloxacin	Clindamycin	Erythromycin	Gentamicin	Rifampicin	Tetracycline	Sulfamethoxazole-Trimethoprim	Multiple Drug Resistance
	C	CIP	CC	E	GM	RA	T	S/T	(MDR)
First chicken shed (*n =* 5)	R	R	R	R	S	S	R	I	C-CIP-CC-E-T
	R	R	R	R	S	S	R	I	C-CIP-CC-E-T
	R	R	R	R	S	S	R	I	C-CIP-CC-E-T
	R	R	R	R	S	S	R	I	C-CIP-CC-E-T
	R	R	R	R	S	S	R	I	C-CIP-CC-E-T
Second chicken shed (*n =* 5)	R	R	R	R	S	S	R	I	C-CIP-CC-E-T
	R	R	R	R	S	S	R	I	C-CIP-CC-E-T
	R	R	R	R	S	S	R	I	C-CIP-CC-E-T
	R	R	R	S	S	S	R	S	C-CIP-CC-T
	R	R	R	R	S	S	R	I	C-CIP-CC-E-T
Exposure square (*n =* 5)	R	R	R	R	S	S	R	S	C-CIP-CC-E-T
	R	R	R	R	S	S	R	S	C-CIP-CC-E-T
	R	R	R	R	S	S	R	S	C-CIP-CC-E-T
	R	R	R	R	S	S	R	I	C-CIP-CC-E-T
	R	R	R	R	S	S	R	I	C-CIP-CC-E-T
3 m Downwind (*n =* 3)	R	R	R	R	S	S	R	I	C-CIP-CC-E-T
	R	R	R	R	S	S	R	I	C-CIP-CC-E-T
	R	R	R	R	S	S	R	I	C-CIP-CC-E-T
5 m Downwind (*n =* 2)	R	R	R	R	S	S	R	I	C-CIP-CC-E-T
	R	R	R	R	S	S	R	I	C-CIP-CC-E-T
20 m Downwind (*n =* 0)	ND	ND	ND	ND	ND	ND	ND	ND	ND
50 m Downwind (*n =* 5)	R	R	R	R	S	S	R	I	C-CIP-CC-E-T
50m Upwind (*n =* 0)	ND	ND	ND	ND	ND	ND	ND	ND	ND
Total percentage (*n =* 21)	21 (100%)	21 (100%)	21 (100%)	20 (95.23%)	0 (0%)	0 (0%)	21 (100%)	0 (0%)	21 (100%)

R = resistance, I = intermediate, S = susceptible; C-CIP-CC-E-T = chloramphenicol–ciprofloxacin–clindamycin–erythromycin–tetracycline; C-CIP-CC-T = chloramphenicol–ciprofloxacin–clindamycin–tetracycline; ND = not detected (sample has no MRSA isolates).

## Data Availability

The data presented in this study are available on request from the corresponding author.

## Supplementary Materials

The following are available online at https://www.mdpi.com/article/10.3390/antibiotics11010081/s1, Table S1. The details of bioaerosol sampling of chicken farm environment; Table S2. MRSA strain identification, *Spa* typing, SCC*mec* typing, and virulence factors detecting genes primers list and PCR conditions; Table S3. Pearson correlation coefficient (r) value and level of significant for environmental parameters and total bacteria count; Table S4. Standard score of zone of inhibition diameter measurements in the disk diffusion method to determine the microbial resistance properties; Table S5. The results of chi-squared tests between sampling point with MRSA strain characterization. References [47,48,49,50,51,52,53] are citied in the Supplementary Materials.
