# Supplementary material for: Role of Bioaerosols on the Short-Distance Transmission of Multidrug-Resistant Methicillin-Resistant Staphylococcus aureus (MRSA) in a Chicken Farm Environment
Source: Antibiotics (Basel). 2022 Jan 10;11(1):81. doi: 10.3390/antibiotics11010081 (PMC8773248; doi:10.3390/antibiotics11010081)
Supplement: Supplementary file 1 [file antibiotics-11-00081-s001.zip › antibiotics-1485000-supplementary.pdf]

antibiotics

Article

Role of Bioaerosols on the Short-Distance Transmission of Multidrug-Resistant Methicillin-Resistant *Staphylococcus aureus* (MRSA) in a Chicken Farm Environment

Bing-Mu Hsu [1,†], Jung-Sheng Chen [2], Gwo-Jong Hsu [3,†], Suprokash Koner [1,4,†], Viji Nagarajan [1] and Hsin-Chi Tsai [5,6,*]

[1] Department of Earth and Environmental Sciences, National Chung Cheng University, Chiayi County 621, Taiwan; bmhsu@eq.ccu.edu.tw (B.-M.H.); suprokashkoner22@gmail.com (S.K.); mathumitha08@gmail.com (V.N.)
[2] Department of Medical Research, E-Da Hospital, Kaohsiung City 824, Taiwan; nicky071214@gmail.com
[3] Division of Infectious Diseases, Ditmanson Medical Foundation, Chia-Yi Christian Hospital, Chiayi City 600, Taiwan; cych01347@gmail.com
[4] Department of Biomedical Sciences, National Chung Cheng University, Chiayi County 621, Taiwan
[5] Department of Psychiatry, School of Medicine, Tzu Chi University, Hualien County 970, Taiwan
[6] Department of Psychiatry, Tzu-Chi General Hospital, Hualien County 970, Taiwan
* Correspondence: cssbmw45@gmail.com; Tel.: +886-38561825
† These authors contributed equally to this work.

Supplementary Materials

Table S1. The details of bioaerosol sampling of chicken farm environment.

Sampling point	Global positioning system (GPS)	Samples type	Air sampling duration	Air flow speed	Total Volume of air sampling
1st chicken shed	23°58'73.80″ N, 120°49'09.34″ E	Bioaerosol	10 min	28.3 (L/min)	283 Litter
2nd chicken shed	23°58'78.32″ N, 120°49'07.84″ E	Bioaerosol	10 min	28.3 (L/min)	283 Litter
Exposure square	23°58'75.76″ N, 120°49'07.95″ E	Bioaerosol	10 min	28.3 (L/min)	283 Litter
3m Downwind	23°58'76.18″ N, 120°49'08.88″ E	Bioaerosol	10 min	28.3 (L/min)	283 Litter
5m Downwind	23°58'76.31″ N, 120°49'10.00″ E	Bioaerosol	10 min	28.3 (L/min)	283 Litter
20m Downwind	23°58'63.17″ N, 120°49'11.04″ E	Bioaerosol	10 min	28.3 (L/min)	283 Litter
50m Downwind	23°58'58.66″ N, 120°49'16.07″ E	Bioaerosol	10 min	28.3 (L/min)	283 Litter
50m Upwind	23°58'78.25″ N, 120°48'93.12″ E	Bioaerosol	10 min	28.3 (L/min)	283 Litter

Table S2. MRSA strain identification, *Spa* typing, *SCCmec* typing, and virulence factors detecting genes primers list and PCR conditions.

Target gene	Size	Sequence (5′ to 3′)	Reaction Materials Final Volume: 25 µl	PCR Condition	Reference
nuc<br>*mecA*	270<br>448	nuc-F 5′-GCGATTGATGGTGATACGGTT-3′<br>nuc-R 5′-AGCCAAGCCTTGACGAACTAAAGC-3′<br>mecA-F 5′-CTCAGGTACTGCTATCCACC-3′<br>mecA-R 5′-CACTTGGTATATCTTCACC-3′	DNA: 100-300 ng<br>Primer: 400 nM nuc FR & mecA FR<br>Master mix: 5 µl	Pre-denaturation: 95°C 5 min<br>Denaturation: 94°C 60s<br>Annealing: 55°C 60s<br>Extension: 72°C 60s<br>D.A.E. Cycles: 30 cycles<br>Final extension: 72°C 10 min	[1,2]

Spa	270	Spa-1113F: 5′- TAAAGACGATCCTTCGGTGAGC -3′<br>Spa-1514R: 5′- CAGCAGTAGTGCCGTTTGCTT -3′	DNA: 100-300 ng<br>Primer: 200 nM Spa FR<br>Master mix: 5 µl	Pre-denaturation: 80°C 5 min<br>Denaturation: 94°C 45s<br>Annealing: 60°C 45s<br>Extension: 72°C 90s<br>D.A.E. Cycles: 35 cycles<br>Final extension: 72°C 10 min	https://spaserver.ridom.de/
SCCmec I<br>SCCmec II<br>SCCmec II, III<br>SCCmec III<br>SCCmec III<br>SCCmec I, II, IV	495<br>284<br>209<br>243<br>414<br>342	CIF2 F2: 5′-TTCGAGTTGCTGATGAAGAAGG-3′<br>CIF2 R2: 5′-ATTTACCACAAGGACTACCAGC-3′<br>KDP F1: 5′-AATCATCTGCCATTGGTGATGC-3′<br>KDP R1: 5′-CGAATGAAGTGAAAGAAAGTGG-3′<br>MECI P2: 5′-ATCAAGACTTGCATTCAGGC-3′<br>MECI P3: 5′-GCGGTTTCAATTCACTTGTC-3′<br>RIF F3: 5′-GTGATTGTTCGAGATATGTGG-3′<br>RIF R9: 5′-CGCTTTATCTGTATCTATCGC-3′<br>RIF F10: 5′-TTCTTAAGTACACGCTGAATCG-3′<br>RIF R13: 5′-GTCACAGTAATTCCATCAATGC-3′<br>DCS F2: 5′-CATCCTATGATAGCTTGGTC-3′<br>DCS R1: 5′-CTAAATCATAGCCATGACCG-3′	DNA: 100-300 ng<br>Primer:<br>400 nM CIF-FR,<br>200 nM KDP-FR,<br>200 nM RIFF3R9<br>400 nM MECI-FR,<br>400 nM RIFF10R13,<br>800 nM DCS-FR<br>Master mix: 5 µl	Pre-denaturation: 94°C 4 min<br>Denaturation: 94°C 30s<br>Annealing: 53°C 30s<br>Extension: 72°C 1 min<br>D.A.E. Cycles: 30 cycles<br>Final extension: 72°C 4 min	[3]
SCCmec V	325	Type V-F: 5′-GAACATTGTTACTTAAATGAGCG-3'<br>Type V-R: 5′-TGAAAGTTGTACCCTTGACACC-3'	DNA: 100-300 ng<br>Primer:<br>100 nM V-FR<br>Master mix: 5 µl	Pre-denaturation: 94°C 5 min<br>Denaturation-1: 94°C 45s<br>Annealing-1: 65°C 45s<br>Extension-1: 72°C 1.5 min<br>D.A.E.-1 Cycles: 10 cycles<br>Denaturation-2: 94°C 45s<br>Annealing-2: 55°C 45s<br>Extension-2: 72°C 1.5 min<br>D.A.E.-2 Cycles: 25 cycles<br>Final extension: 72°C 10 min	[4]
SCCmec VI	134	ccrB4-F: 5′-CGAAGTATAGACACTGGAGCGATA-3′<br>ccrB4-R: 5′-GCGACTCTCTTGGCGTTTA-3′	DNA: 100-300 ng<br>Primer:<br>100 nM FR,<br>Master mix: 5 µl	Pre-denaturation: 95°C 10 min<br>Denaturation: 95°C 30s<br>Annealing: 50°C 30s<br>Extension: 72°C 30s<br>D.A.E. Cycles: 40 cycles<br>Final extension: 72°C 5 min	[5]

SCCmec VII<br>SCCmec VIII	473<br>138	Type VII F: 5′-GTGACGTTGATATTGCAGTGGT-3′<br>Type VII R: 5′-TGAAGAAGTTTGTTCCGCGT-3′<br>Type VIII F: 5′-AGCGACGATGAACAACACCGCTACTTACTCAA-3′<br>Type VIII R: 5′-TTGGTTGAGAATGAGAACAGTGGTAAGATC-3′	DNA: 100-300 ng<br>Primer: 400 nM FR<br>Master mix: 5 µl	Pre-denaturation: 95°C 2 min<br>Denaturation: 95°C 30s<br>Annealing: 54°C 1 min<br>Extension: 72°C 1 min 20s<br>D.A.E. Cycles: 35 cycles<br>Final extension: 72°C 7 min	[6]
PVL	433	PVL-1: 5′-ATCATTAGGTAAAATGTCTGGACATGATCCA-3'<br>PVL-2: 5′-GCATCAAGTGTATTGGATAGCAAAAGC-3′	DNA: 100-300 ng<br>Primer: 400 nM FR<br>Master mix: 5 µl	Pre-denaturation: 94°C 5 min<br>Denaturation: 94°C 40s<br>Annealing: 53°C 40s<br>Extension: 72°C 1 min<br>D.A.E. Cycles: 35 cycles<br>Final extension: 72°C 10 min	[7]
entA<br>*entB*<br>*entC*<br>*entD*<br>*entE*<br>*tsst-1*<br>*eta*<br>*etb*	121<br>478<br>459<br>384<br>495<br>271<br>464<br>200	entA-F: 5′-TTGGAACGGTTAAAACGAA-3′<br>entA-R: 5′-GAACCTTCCCATCAAAAACA-3′<br>entB-F: 5′-TCGCATCAAACTGACAAACG-3′<br>entB-R: 5′-GCAGGTACTCTATAAGTGCC-3′<br>entC-F: 5′-GGAGGAATAACAAAACATGAAGG-3′<br>entC-R: 5′-AAAGGCAAGCACCGAAGTAC-3′<br>entD-F: 5′-TGGTGGTGAAATAGATAGGAC-3′<br>entD-R: 5′-TGAAGGTGCTCTGTGGATAAT-3′<br>entE-F: 5′-TGGTAGCGAGAAAAGCGAAG-3′<br>entE-R: 5′-TGTAAATAATGCCTTGCCTGAA-3′<br>tsst-1-F: 5′-CTGGTATAGTAGTGGGTCTG-3′<br>tsst-1-R: 5′-AGGTAGTTCTATTGGAGTAGG-3′<br>eta-F: 5′-TTTGCTTTCTTGATTTGGATTC-3′<br>eta-R: 5′-GATGTGTTCGGTTTGATTGAC-3′<br>etb-F: 5′-ACGGCTATATACATTCAATT-3′<br>etb-R: 5′-TCCATCGATAATATACCTAA-3′	DNA: 100-300 ng<br>Primer: 400 nM Primer FR<br>Master mix: 5 µl	Pre-denaturation: 94°C 5 min<br>Denaturation: 94°C 1 min<br>Annealing: 2 min<br>Extension: 72°C 1 min<br>D.A.E. Cycles: 35 cycles<br>Final extension: 72°C 5 min<br>AnnealingTemp.<br>*entA*: 50°C<br>*entB*: 55°C<br>*entC*: 59°C<br>*entD*: 51°C<br>*entE*: 55.5°C<br>*tsst-1*: 54°C<br>*eta*: 54°C<br>*etb*: 50.9°C	[8]

Table S3. Pearson correlation coefficient (r) value and level of significant for environmental parameters and total bacteria count.

	**Correlations test**				
	**Ammonia**	**Methylamine**	**Sampling Distance**	**Wind speed (m/s)**	**Total bacteria count**
Ammonia	1.000	0.961**	-0.092	-0.447	0.865**
Methylamine	0.961**	1.000	-0.089	-0.443	0.940**
Sampling Distance	-0.092	-0.089	1.000	-0.490	-0.048
Wind speed (m/s)	-0.447	-0.443	-0.490	1.000	-0.476
Total bacteria count	0.865**	0.940**	-0.048	-0.476	1.000

**. Correlation is significant at the 0.01 level (2-tailed).

Table S4. Standard score of Zone of inhibition diameter measurement in disk diffusion method to determine the microbial resistance property.

**Antibiotic name**	**Zone of inhibition diameter measurement value (mm)**		
	**Resistant**	**Intermediate**	**Susceptible**
Chloramphenicol	≦12	13-17	≧18
Ciprofloxacin	≦15	16-20	≧21
Clindamycin	≦14	15-20	≧21
Erythromycin	≦13	14-22	≧23
Gentamicin	≦12	13-14	≧15
Rifampicin	≦16	17-19	≧20
Tetracycline	≦14	15-18	≧19
Sulfamethoxazole-Trimethoprim	≦10	11-15	≧16

A reference documents CLSI M100-S27 which is modified in 2017 has been used to determine the score of Zone of inhibition diameter measurement [9]

Table S5. The results of Chi-square test between sampling point with MRSA strain characterization

**Chi-Square test**			
	**Sampling point**		
Variables	Degree of freedom (df)	Pearson Chi-Square	Asymptotic Significance (2-sided)
SCCmec typing	7	23.000[a]	0.002
MRSA grouping	7	23.000[a]	0.002
Spa type	7	23.000[a]	0.002
Virulence factors	21	53.959[b]	0.000
Multiple drug resistance	14	26.680[c]	0.021

a. 16 cells (100.0%) have expected count less than 5. The minimum expected count is .09.
b. 32 cells (100.0%) have expected count less than 5. The minimum expected count is .09.
c. 24 cells (100.0%) have expected count less than 5. The minimum expected count is .04.

References

1. Brakstad, O.G.; Aasbakk, K.; Maeland, J.A. Detection of Staphylococcus aureus by polymerase chain reaction amplification of the nuc gene. *Journal of Clinical Microbiology* **1992**, *30*, 1654-1660, doi:doi:10.1128/jcm.30.7.1654-1660.1992.
2. Sakoulas, G.; Gold, H.S.; Venkataraman, L.; DeGirolami, P.C.; Eliopoulos, G.M.; Qian, Q. Methicillin-resistant Staphylococcus aureus: comparison of susceptibility testing methods and analysis of mecA-positive susceptible strains. *Journal of clinical microbiology* **2001**, *39*, 3946-3951.
3. Oliveira, D.C.; Lencastre, H.d. Multiplex PCR Strategy for Rapid Identification of Structural Types and Variants of the <i>mec</i> Element in Methicillin-Resistant <i>Staphylococcus aureus</i>. *Antimicrobial Agents and Chemotherapy* **2002**, *46*, 2155-2161, doi:doi:10.1128/AAC.46.7.2155-2161.2002.
4. Zhang, K.; McClure, J.-A.; Elsayed, S.; Louie, T.; Conly, J.M. Novel Multiplex PCR Assay for Characterization and Concomitant Subtyping of Staphylococcal Cassette Chromosome <i>mec</i> Types I to V in Methicillin-Resistant <i>Staphylococcus aureus</i>. *Journal of Clinical Microbiology* **2005**, *43*, 5026-5033, doi:doi:10.1128/JCM.43.10.5026-5033.2005.
5. Chen, L.; Mediavilla José, R.; Oliveira Duarte, C.; Willey Barbara, M.; de Lencastre, H.; Kreiswirth Barry, N. Multiplex Real-Time PCR for Rapid Staphylococcal Cassette Chromosome mec Typing. *Journal of Clinical Microbiology* **2009**, *47*, 3692-3706, doi:10.1128/JCM.00766-09.
6. Bhowmik, D.; Das, B.J.; Pandey, P.; Chetri, S.; Chanda, D.D.; Bhattacharjee, A. An array of multiplex PCR assays for detection of staphylococcal chromosomal cassette mec (SCCmec) types among staphylococcal isolates. *Journal of microbiological methods* **2019**, *166*, 105733.
7. Asghar, A.H. Molecular characterization of methicillin-resistant Staphylococcus aureus isolated from tertiary care hospitals. *Pak J Med Sci* **2014**, *30*, 698-702, doi:10.12669/pjms.304.4946.
8. Imani Fooladi, A.A.; Ashrafi, E.; Tazandareh, S.G.; Koosha, R.Z.; Rad, H.S.; Amin, M.; Soori, M.; Larki, R.A.; Choopani, A.; Hosseini, H.M. The distribution of pathogenic and toxigenic genes among MRSA and MSSA clinical isolates. *Microbial Pathogenesis* **2015**, *81*, 60-66, doi:https://doi.org/10.1016/j.micpath.2015.03.013.
9. Clinical; Institute, L.S. Performance standards for antimicrobial susceptibility testing. **2017**.
